# On Singleton Arc Consistency for CSPs Defined by Monotone Patterns

**DOI:** 10.1007/s00453-018-0498-2

**Published:** 2018-08-13

**Authors:** Clément Carbonnel, David A. Cohen, Martin C. Cooper, Stanislav Živný

**Affiliations:** 1 University of Oxford, Oxford, UK; 2 Royal Holloway, University of London, London, UK; 3 IRIT, University of Toulouse III, Toulouse, France

**Keywords:** Constraint satisfaction problems, Forbidden patterns, Singleton arc consistency

## Abstract

Singleton arc consistency is an important type of local consistency which has been recently shown to solve all constraint satisfaction problems (CSPs) over constraint languages of bounded width. We aim to characterise all classes of CSPs defined by a forbidden pattern that are solved by singleton arc consistency and closed under removing constraints. We identify five new patterns whose absence ensures solvability by singleton arc consistency, four of which are provably maximal and three of which generalise 2-SAT. Combined with simple counter-examples for other patterns, we make significant progress towards a complete classification.

## Introduction

The constraint satisfaction problem (CSP) is a declarative paradigm for expressing computational problems. An instance of the CSP consists of a number of variables to which we need to assign values from some domain. Some subsets of the variables are constrained in that they are not permitted to take all values in the product of their domains. The scope of a constraint is the set of variables whose values are limited by the constraint, and the constraint relation is the set of permitted assignments to the variables of the scope. A solution to a CSP instance is an assignment of values to variables in such a way that every constraint is satisfied, i.e. every scope is assigned to an element of the constraint relation.

The CSP has proved to be a useful technique for modelling in many important application areas from manufacturing to process optimisation, for example planning and scheduling optimisation [31], resource allocation [29], job shop problems [14] and workflow management [33]. Hence much work has been done on describing useful classes of constraints [3] and implementing efficient algorithms for processing constraints [7]. Many constraint solvers use a form of backtracking where successive variables are assigned values that satisfy all constraints. In order to mitigate the exponential complexity of backtracking some form of pre-processing is always performed. These pre-processing techniques identify values that cannot be part of any solution in an effective way and then propagate the effects of removing these values throughout the problem instance. Of key importance amongst these pre-processing algorithms are the relatives of arc consistency propagation including generalised arc consistency (GAC) and singleton arc consistency (SAC). Surprisingly there are large classes [13, 16, 23, 28] of the CSP for which GAC or SAC are decision procedures: after establishing consistency if every variable still has a non-empty domain then the instance has a solution.

More generally, these results fit into the wider area of research aiming to identify sub-problems of the CSP for which certain polynomial-time algorithms are decision procedures. Perhaps the most natural ways to restrict the CSP is to limit the constraint relations that we allow or to limit the structure of (the hypergraph of) interactions of the constraint scopes. A set of allowed constraint relations is called a constraint language. A subset of the CSP defined by limiting the scope interactions is called a structural class.

There has been considerable success in identifying tractable constraint languages, recently yielding a full classification of the complexity of finite constraint languages [11, 34]. Techniques from universal algebra have been essential in this work as the complexity of a constraint language is characterised by a particular algebraic structure [10]. The two most important algorithms for solving the CSP over tractable constraint languages are local consistency and the few subpowers algorithm [9, 27], which generalises ideas from group theory. A necessary and sufficient condition for solvability by the few subpowers algorithm was identified in [4, 27]. The set of all constraint languages decided by local consistency was later described by Barto and Kozik [2] and independently by Bulatov [8]. Surprisingly, all such languages are in fact decided by establishing singleton arc consistency [28].

A necessary condition for the tractability of a structural class with bounded arity is that it has bounded treewidth modulo homomorphic equivalence [26]. In all such cases we decide an instance by establishing *k*-consistency, where *k* is the treewidth of the core. It was later shown that the converse holds: if a class of structures does not have treewidth *k* modulo homomorphic equivalence then it is not solved by *k*-consistency [1], thus fully characterising the strength of consistency algorithms for structural restrictions. Both language-restricted CSPs and CSPs of bounded treewidth are *monotone* in the sense that we can relax (remove constraints from) any CSP instance without affecting its membership in such a class.

Since our understanding of consistency algorithms for language and structural classes is so well advanced there is now much interest in so called hybrid classes, which are neither definable by restricting the language nor by limiting the structure. For the binary CSP, one popular mechanism for defining hybrid classes follows the considerable success of mapping the complexity landscape for graph problems in the absence of certain induced subgraphs or graph minors. Here, hybrid (binary) CSP problems are defined by forbidding a fixed set of substructures (*patterns*) from occurring in the instance [17]. This framework is particularly useful in algorithm analysis, since it allows us to identify precisely the local properties of a CSP instance that make it impossible to solve via a given polynomial-time algorithm. This approach has recently been used to obtain a pattern-based characterisation of solvability by arc consistency [23], a detailed analysis of variable elimination rules [15] and various novel tractable classes of CSP [19, 20].

Singleton arc consistency is a prime candidate to study in this framework since it is one of the most prominent incomplete polynomial-time algorithms for CSP and the highest level of consistency (among commonly studied consistencies) that operates only by removing values from domains. This property ensures that enforcing SAC cannot introduce new patterns, which greatly facilitates the analysis. It is therefore natural to ask for which patterns, forbidding their occurrence ensures that SAC is a sound decision procedure. In this paper we make a significant contribution towards this objective by identifying five patterns which define classes of CSPs decidable by SAC. All five classes are monotone, and we show that only a handful of open cases separates us from an essentially full characterisation of monotone CSP classes decidable by SAC and definable by a forbidden pattern. Some of our results rely on a novel proof technique which follows the *trace* of a successful run of the SAC algorithm to dynamically identify redundant substructures in the instance and construct a solution.

The structure of the paper is as follows. In Sect. 2 we provide essential definitions and background theory. In Sect. 3 we state the main results. In Sect. 4 we introduce the trace technique, which is then used in Sects. 5 and 6 to establish the tractability of three patterns from our main result. The tractability of the remaining two patterns from the main result is shown in Sect. 7. In Sect. 8 we give a necessary condition for the solvability by SAC. Finally, we conclude the paper in Sect. 9 with some open problems.

## Preliminaries

*CSP* A *binary CSP instance* is a triple $I=(X,D,C)$, where *X* is a finite set of variables, *D* is a finite domain, each variable $x\in X$ has its own domain of possible values $D(x) \subseteq D$, and $C=\{R(x,y)\mid x, y\in X, x\ne y\}$, where $R(x,y)\subseteq D^2$, is the set of constraints. We assume, without loss of generality, that each pair of variables $x,y \in X$ is constrained by a constraint *R*(*x*, *y*). (Otherwise we set $R(x,y)=D(x) \times D(y)$.) We also assume that $(a,b) \in R(x,y)$ if and only if $(b,a) \in R(y,x)$. A constraint is *trivial* if it contains the Cartesian product of the domains of the two variables. By *deleting* a constraint we mean replacing it with a trivial constraint. The *projection*$I[X']$ of a binary CSP instance *I* on $X'\subseteq X$ is obtained by removing all variables in $X \backslash X'$ and all constraints *R*(*x*, *y*) with $\{x,y\} \not \subseteq X'$. A *partial solution* to a binary CSP instance on $X'\subseteq X$ is an assignment *s* of values to variables in $X'$ such that $s(x) \in D(x)$ for all $x \in X'$ and $(s(x),s(y)) \in R(x,y)$ for all constraints *R*(*x*, *y*) with $x,y \in X'$. A *solution* to a binary CSP instance is a partial solution on *X*.

An assignment (*x*, *a*) is called a *point*. For simplicity of notation we can assume that variable domains are disjoint, so that using *a* as a shorthand for (*x*, *a*) is unambiguous. If $(a,b) \in R(x,y)$, we say that the assignments (*x*, *a*), (*y*, *b*) (or more simply *a*, *b*) are *compatible* and that *ab* is a *positive edge*, otherwise *a*, *b* are *incompatible* and *ab* is a *negative edge*. We say that $a \in D(x)$ has a *support* at variable *y* if $\exists b \in D(y)$ such that *ab* is a positive edge.

The constraint graph of a CSP instance with variables *X* is the graph $G=(X,E)$ such that $(x,y)\in E$ if *R*(*x*, *y*) is non-trivial. The *degree* of a variable *x* in a CSP instance is the degree of *x* in the constraint graph of the instance.

*Arc Consistency* A domain value $a \in D(x)$ is *arc consistent* if it has a support at every other variable. A CSP instance is *arc consistent* (AC) if every domain value is arc consistent.

*Singleton Arc Consistency* Singleton arc consistency is stronger than arc consistency (but weaker than strong path consistency [30]). A domain value $a \in D(x)$ in a CSP instance *I* is *singleton arc consistent* if the instance obtained from *I* by removing all domain values $b \in D(x)$ with $a\ne b$ can be made arc consistent without emptying any domain. A CSP instance is *singleton arc consistent* (SAC) if every domain value is singleton arc consistent.

*Establishing Consistency* Domain values that are not arc consistent or not singleton arc consistent cannot be part of a solution so can safely be removed. The closure of a CSP instance under the removal of values that are not (singleton) arc consistent is unique, and the process of reducing an instance to its closure is called *establishing (singleton) arc consistency* [32]. For a binary CSP instance with domain size *d*, *n* variables and *e* non-trivial constraints there are $O(ed^2)$ algorithms for establishing arc consistency [6] and $O(ned^3)$ algorithms for establishing singleton arc consistency [5].

SAC *decides* a CSP instance if, after establishing singleton arc consistency, non-empty domains for all variables guarantee the existence of a solution. SAC decides a class of CSP instances if SAC decides every instance from the class.

*Neighbourhood Substitutability* If $a,b \in D(x)$, then *a* is *neighbourhood substitutable* (or is *dominated*) by *b* if there is no *c* such that *ac* is a positive edge and *bc* a negative edge: such values *a* can be deleted from *D*(*x*) without changing the satisfiability of the instance since *a* can be replaced by *b* in any solution [25]. Similarly, removing neighbourhood substitutable values cannot destroy (singleton) arc consistency.

*Patterns* In a binary CSP instance each constraint decides, for each pair of values in *D*, whether it is allowed. Hence a binary CSP can also be defined as a set of points $X\times D$ together with a compatibility function that maps each edge, (*x*, *a*)(*y*, *b*) with $x \ne y$, into the set $\{\text{ negative },\text{ positive }\}$. A *pattern* extends the notion of a binary CSP instance by allowing the compatibility function to be partial. A pattern *Poccurs* (as a subpattern) in an instance *I* if there is mapping from the points of *P* to the points of *I* which respects variables (two points are mapped to points of the same variable in *I* if and only if they belong to the same variable in *P*) and maps negative edges to negative edges, and positive edges to positive edges. A set of patterns occurs in an instance *I* if at least one pattern in the set occurs in *I*.

We use the notation $\hbox {CSP}(\overline{P})$ for the set of binary instances in which *P* does not occur as a subpattern. A pattern *P* is *SAC-solvable* if SAC decides $\hbox {CSP}(\overline{P})$. It is worth observing that $\hbox {CSP}(\overline{P})$ is closed under the operation of establishing (singleton) arc consistency. A pattern *P* is *tractable* if $\hbox {CSP}(\overline{P})$ can be solved in polynomial time.

Points (*x*, *a*) and (*x*, *b*) in a pattern are *mergeable* if there is no point (*y*, *c*) such that *ac* is positive and *bc* is negative or vice versa. For each set of patterns there exists a set of patterns without mergeable points which occur in the same set of instances.

A point (*x*, *a*) in a pattern is called *dangling* if there is at most one *b* such that *ab* is a positive edge and no *c* such that *ac* is a negative edge. Dangling points are redundant when considering the occurrence of a pattern in an arc consistent CSP instance.

A pattern is called *irreducible* if it has no dangling points and no mergeable points [20]. When studying algorithms that are at least as strong as arc consistency, a classification with respect to forbidden *sets* of irreducible patterns is equivalent to a classification with respect to all forbidden sets of patterns. For this reason classifications are often established with respect to irreducible patterns even if only classes definable by forbidding a single pattern are considered [20, 23], as we do in the present paper.

## Results

Call a class $\mathcal {C}$ of CSP instances *monotone* if deleting any constraint from an instance $I \in \mathcal {C}$ produces another instance in $\mathcal {C}$. For example, language classes and bounded treewidth classes are monotone. An interesting research direction is to study those monotone classes defined by a forbidden pattern which are solved by singleton arc consistency, both in order to uncover new tractable classes and to better understand the strength of SAC.

We call a pattern *monotone* if when forbidden it defines a monotone class. Monotone patterns can easily be seen to correspond to exactly those patterns in which positive edges only occur in constraints which have at least one negative edge. To see this, firstly let *P* be a pattern in which positive edges only occur in constraints which have at least one negative edge. Note that deleting a constraint in an instance *I* cannot introduce *P*, so $\hbox {CSP}(\overline{P})$ is monotone. To see the converse, let *Q* be a pattern in which a positive edge *e* occurs in a constraint *c* with no negative edges. Let $Q'$ be equivalent to *Q* but with *e* replaced by a negative edge. Let $I'$ be the instance obtained by completing $Q'$ with negative edges (i.e. joining by negative edges all pairs of points at different variables whose compatibility is unspecified in $Q'$). Let $I'[-c]$ be the instance $I'$ in which the constraint (corresponding to) *c* has been deleted. Now *Q* occurs in $I'[-c]$ (since the positive edge *e* has been reintroduced by deleting *c*) but not in $I'$ (which can be seen by simply counting the number of constraints containing positive edges). Thus $\hbox {CSP}(\overline{Q})$ is not monotone.

**Fig. 1:**
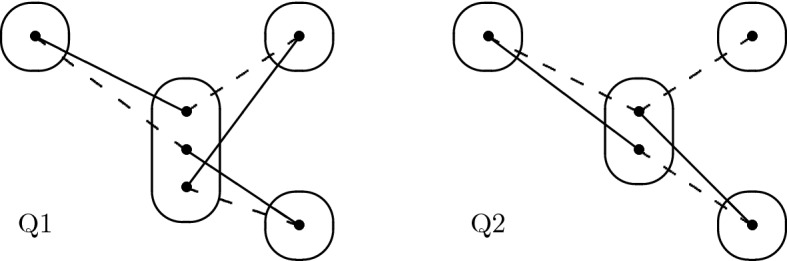
All degree-3 irreducible monotone patterns solved by SAC must occur in at least one of these patterns

**Fig. 2:**
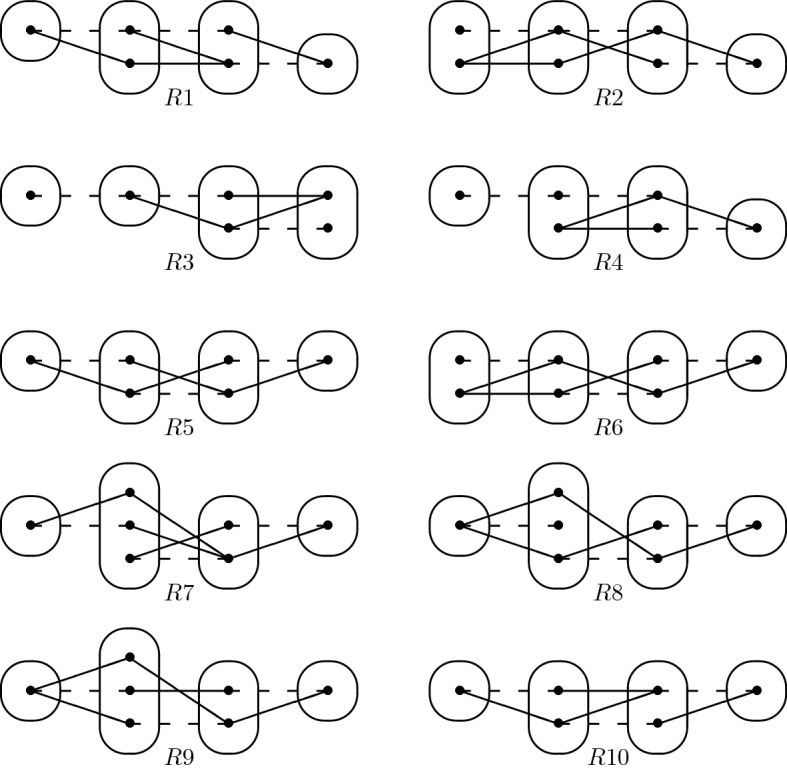
All degree-2 irreducible monotone patterns solved by SAC must occur in at least one of these patterns

**Fig. 3:**
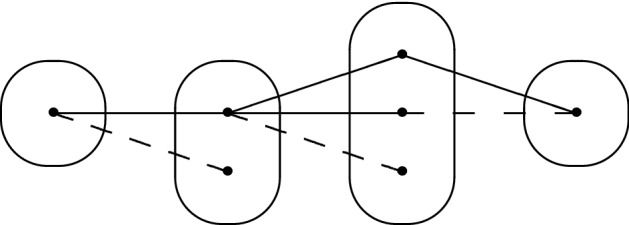
The pattern R7-, a subpattern of R7

Consider the monotone patterns Q1 and Q2 shown in Fig. 1, patterns R5, R8 shown in Fig. 2, and pattern R7- shown in Fig. 3.

### Theorem

(Main) The patterns Q1, Q2, R5, R8, and R7- are SAC-solvable.

In order to prove the SAC-solvability of Q1, R8 and R7- we use the same idea of following the trace of arc consistency and argue that the resulting instance is not too complicated. While the same idea is behind the proofs of all three patterns, the technical details differ.

In the remaining two cases we identify an operation that preserves SAC and satisfiability, does not introduce the pattern and after repeated application necessarily produces an equivalent instance which is solved by SAC. In the case of R5, the operation is simply removing any constraint. In the case of Q2, the operation is BTP-merging [19].

### Remark 1

By Proposition 2 from Sect. 8, any *monotone* and *irreducible* pattern solvable by SAC must occur in at least one of the patterns shown in Figs. 1 and 2. By this analysis, we have managed to reduce the number of remaining cases to a handful. Our main result shows that some of these are SAC-solvable. In particular, the patterns Q1, Q2, R5, and R8 are maximal in the sense that adding anything to them would give a pattern that is either non-monotone or not solved by SAC.

### Remark 2

**Fig. 4:**
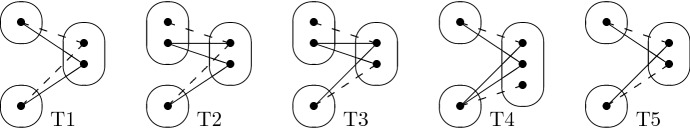
The set of tractable 2-constraint irreducible patterns

We point out that certain interesting forbidden patterns, such as BTP [21], NegTrans [22], and EMC [23] are not monotone. On the other hand, the patterns T1$,\ldots ,$T5 shown in Fig. 4*are* monotone. Patterns T1$,\ldots ,$T5 were identified in [20] as the maximal irreducible tractable patterns on two connected constraints. We show in Sect. 8 that T1 is not solved by SAC. Our main result implies (since R8 contains T4 and T5) that both T4 and T5 are solved by SAC. It can easily be shown, from Lemma 9 and [20, Lemma 25], that T2 is solved by SAC, and we provide, in “Appendix”, a simple proof that T3 is solved by SAC as well. Hence, we have characterised all 2-constraint irreducible patterns solvable by SAC.

### Remark 3

Observe that Q1 does not occur in any binary CSP instance in which all degree 3 or more variables are Boolean. This shows that 2-SAT is a strict subset of CSP($\overline{\hbox {Q1}}$). This class is incomparable with language-based generalisations of 2-SAT, such as the class ZOA [18] (the language of “zero-one-all” relations, that is, of all relations that admit the dual discriminator polymorphism) since in CSP($\overline{\hbox {Q1}}$) degree-2 variables can be constrained by arbitrary constraints. Indeed, instances in CSP($\overline{\hbox {Q1}}$) can have an arbitrary constraint on the pair of variables *x*, *y*, where *x* is of arbitrary degree and of arbitrary domain size if for all variables $z \notin \{x,y\}$, the constraint on the pair of variables *x*, *z* is of the form $(x \in S) \vee (z \in T_z)$ where *S* is fixed (i.e. independent of *z*) but $T_z$ is arbitrary. R8 and R7- generalise T4 and CSP($\overline{\hbox {T4}}$) generalises ZOA [20], so CSP($\overline{\hbox {R8}}$) and CSP($\overline{\hbox {R7-}}$) are strict generalisations of ZOA.

## Notation for the Trace Technique

Given a singleton arc consistent instance *I*, a variable *x* and a value $v \in D(x)$, we denote by $I_{xv}$ the instance obtained by assigning *x* to *v* (that is, setting $D(x) = \{v\}$) and enforcing arc consistency. To avoid confusion with the original domains, we will use $D_{xv}(y)$ to denote the domain of the variable *y* in $I_{xv}$. For our proofs we will assume that arc consistency has been enforced using a straightforward algorithm that examines the constraints one at a time and removes the points that do not have a support until a fixed point is reached. We will be interested in the *trace* of this algorithm, given as a chain of propagations:

$$\begin{aligned} (P_{xv}): (x \rightarrow y_0), (x_1 \rightarrow y_1), (x_2 \rightarrow y_2), \ldots , (x_p \rightarrow y_p) \end{aligned}$$

where $x_i \rightarrow y_i$ means that the algorithm has inferred a change in the domain of $y_i$ when examining the constraint $R(x_i,y_i)$. We define a map $\rho : (P_{xv})\mapsto 2^D$ that maps each $(x_i \rightarrow y_i) \in (P_{xv})$ to the set of values that were removed from $D_{xv}(y_i)$ at this step. Without loss of generality, we assume that the steps $(x_i \rightarrow y_i)$ such that the pruning of $\rho (x_i \rightarrow y_i)$ from $D_{xv}(y_i)$ does not incur further propagation are performed last.

We denote by $S_{(P_{xv})}$ the set of variables that appear in $(P_{xv})$. Because *I* was (singleton) arc consistent before *x* was assigned, we have $S_{(P_{xv})}= \{x\} \cup \{y_i \mid i \ge 0 \}$. We rename the elements of $S_{(P_{xv})}$ as $\{p_i \mid i \ge 0\}$ where the index *i* denotes the order of first appearance in $(P_{xv})$. Finally, we use $S^I_{(P_{xv})}$ to denote the set of *inner* variables, that is, the set of all variables $p_j \in S_{(P_{xv})}$ for which there exists $p_r \in S_{(P_{xv})}$ such that $(p_j \rightarrow p_r) \in (P_{xv})$.

## Tractability of Q1

Consider the pattern Q1 shown in Fig. 1. Let $I \in$ CSP($\overline{\hbox {Q1}}$) be a singleton arc consistent instance, *x* be any variable and *v* be any value in the domain of *x*. Our proof of the SAC-decidability of CSP($\overline{\hbox {Q1}}$) uses the trace of the arc consistency algorithm to determine a subset of variables in the vicinity of *x* such that (*i*) the projection of $I_{xv}$ to this particular subset is satisfiable, (*ii*) those variables do not interact too much with the rest of the instance and (*iii*) the projections of $I_{xv}$ and *I* on the other variables are almost the same. We then use these three properties to show that the satisfiability of *I* is equivalent to that of an instance with fewer variables, and we repeat the operation until the smaller instance is trivially satisfiable.

The following lemma describes the particular structure of $I_{xv}$ around the variables whose domain has been reduced by arc consistency. Note that a non-trivial constraint in *I* can be trivial in $I_{xv}$ because of domain changes; unless otherwise stated the triviality/non-triviality of constraints is always discussed with respect to $I_{xv}$.

### Lemma 1

Consider the instance $I_{xv}$. Every variable $p_i \in S^I_{(P_{xv})}$ is in the scope of at most two non-trivial constraints, which must be of the form $R(p_j,p_i)$ and $R(p_i,p_r)$ with $j < i$, $(p_j \rightarrow p_i) \in (P_{xv})$ and $(p_i \rightarrow p_r) \in (P_{xv})$.

### Proof

The claim is true for $p_0 = x$ as every constraint incident to *x* is trivial. Otherwise, let $p_i \in S^I_{(P_{xv})}$ be such that $p_i \ne x$. Let $p_j$, $j < i$ be such that $(p_j \rightarrow p_i)$ occurs first in $(P_{xv})$. Because $p_i \in S^I_{(P_{xv})}$ and we assumed that the arc consistency algorithm performs the pruning that do not incur further propagation last, we know that there exists $c_i \in \rho (p_j \rightarrow p_i)$ and $p_r \in S_{(P_{xv})}$ with $(p_i \rightarrow p_r) \in (P_{xv})$ such that the pruning of $c_i$ from $D(p_i)$ allows the pruning of some $a_r \in \rho (p_i \rightarrow p_r)$ from the domain of $p_r$. It follows that $(c_i,a_r) \in R(p_i,p_r)$, $(v_i,a_r) \notin R(p_i,p_r)$ for any $v_i \in D_{xv}(p_i)$ and $(v_j,c_i) \notin R(p_j,p_i)$ for any $v_j \in D_{xv}(p_j)$. Moreover, $a_r$ was a support for $c_i$ at $p_r$ when $c_i$ was pruned so we know that $p_j \ne p_r$.

**Fig. 5:**
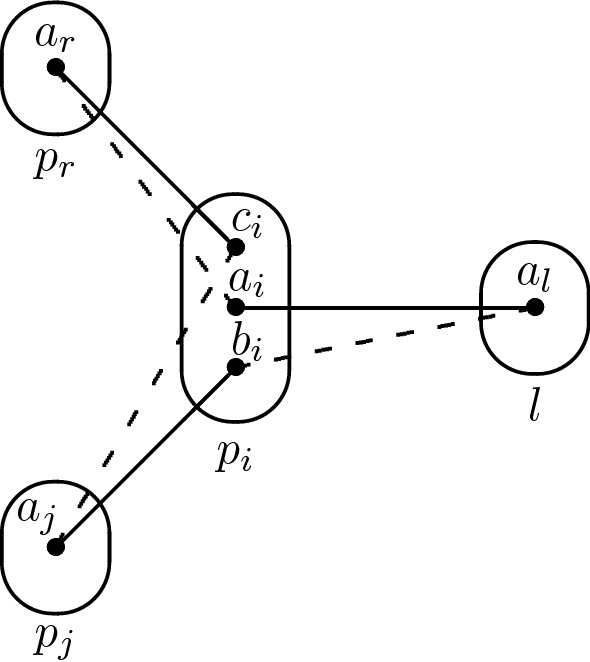
The occurence of Q1 in the proof of Lemma 1

For the sake of contradiction, let us assume that there exists a constraint $R(p_i,l)$ with $l \notin \{p_j,p_r\}$ that is not trivial. In particular, there exist $a_i,b_i \in D_{xv}(p_i)$ and $a_l \in D_{xv}(l)$ such that $(a_i,a_l) \in R(p_i,l)$ but $(b_i,a_l) \notin R(p_i,l)$. Since $a_i$ is in $D_{xv}(p_i)$ and $a_r$ was removed by arc consistency when inspecting the constraint $R(p_i,p_r)$, we have $(a_i,a_r) \notin R(p_i,p_r)$. $I_{xv}$ is arc consistent so there exists some $a_j \in D_{xv}(p_j)$ such that $(a_j,b_i) \in R(p_j,p_i)$, and since $c_i \in \rho (p_j \rightarrow p_i)$ we have $(a_j,c_i) \notin R(p_j,p_i)$. At this point we have reached the desired contradiction as Q1 occurs on $(p_i,p_j,p_r,l)$ with $p_i$ being the middle variable (see Fig. 5). $\square$

Given a subset *S* of variables, an *S*-*path* between two variables $y_1$ and $y_2$ is a path $R(y_1,x_2),R(x_2,x_3),\ldots ,R(x_{k},y_2)$ of non-trivial constraints with $k \ge 2$ and $x_2,\ldots ,x_{k} \in S$.

### Lemma 2

Consider the instance $I_{xv}$. There is no $(S^I_{(P_{xv})})$-path between two variables in $X\backslash S^I_{(P_{xv})}$ and there is no cycle of non-trivial constraints in $I_{xv}[S^I_{(P_{xv})}]$.

### Proof

Let $y_1,y_2 \in X\backslash S^I_{(P_{xv})}$ and assume for the sake of contradiction that a $(S^I_{(P_{xv})})$-path $R(y_1,x_2),R(x_2,x_3),\ldots ,R(x_{k-1},y_2)$ exists. Let $p_i \in \{x_2,\ldots ,x_{k-1}\}$ be such that *i* is minimum. Since $p_i$ is in the scope of two non-trivial constraints in this path, it follows from Lemma 1 that $p_i$ is in the scope of exactly two non-trivial constraints, one of which is of the form $R(p_j,p_i)$ with $j < i$ and $(p_j \rightarrow p_i) \in (P_{xv})$. It follows from $(p_j \rightarrow p_i) \in (P_{xv})$ that $p_j \in S^I_{(P_{xv})}$ and hence $p_j$ is not an endpoint of the path, and then $j < i$ contradicts the minimality of *i*. The second part of the claim follows from the same argument, by considering a cycle as a $(S^I_{(P_{xv})})$-path $R(x_1,x_2),R(x_2,x_3),\ldots ,R(x_{k-1},x_1)$ with $x_1 \in (S^I_{(P_{xv})})$ and defining $p_i$ as the variable among $\{x_1,\ldots ,x_{k-1}\}$ with minimum index. $\square$

### Lemma 3

$I_{xv}$ has a solution if and only if $I_{xv}[X\backslash S^I_{(P_{xv})}]$ has a solution.

### Proof

The “only if” implication is trivial, so we focus on the other direction. Suppose that there exists a solution $\phi$ to $I_{xv}[X\backslash S^I_{(P_{xv})}]$. Let *Y* be a set of variables initialized to $X\backslash S^I_{(P_{xv})}$. We will grow *Y* with the invariants that (*i*) we know a solution $\phi$ to $I_{xv}[Y]$, and (*ii*) there is no $(X\backslash Y)$-path between two variables in *Y* (which is true at the initial state by Lemma 2).

If there is no non-trivial constraint between $X\backslash Y$ and *Y* then $I_{xv}$ is satisfiable if and only if $I_{xv}[X\backslash Y]$ is. By construction $X\backslash Y \subseteq S^I_{(P_{xv})}$ and by Lemma 2 we know that $I_{xv}[X\backslash Y]$ has no cycle of non-trivial constraints. Because $I_{xv}[X\backslash Y]$ is arc consistent and acyclic it has a solution [24], and we can conclude that in this case $I_{xv}$ has a solution.

Otherwise, let $p_i \in X\backslash Y$ be such that there exists a non-trivial constraint between $p_i$ and some variable $y \in Y$. By (*ii*), this non-trivial constraint must be unique (with respect to $p_i$) as otherwise we would have a $(X\backslash Y)$-path between two variables in *Y*. By arc consistency, there exists $a_i \in D_{xv}(p_i)$ such that $(a_i,\phi (y)) \in R(p_i,y)$; because this non-trivial constraint is unique, setting $\phi (p_i) = a_i$ yields a solution to $I_{xv}[Y \cup \{p_i\}]$. Because any $(X\backslash (Y \cup \{p_i\}))$-path between two variables in $Y \cup \{p_i\}$ would extend to a $(X\backslash Y)$-path between *Y* variables by going through $p_i$, we know that no such path exists. Then $Y \leftarrow Y \cup \{p_i\}$ satisfies both invariants, so we can repeat the operation until we have a solution to the whole instance or all constraints between *Y* and $X\backslash Y$ are trivial. In both cases $I_{xv}$ has a solution. $\square$

### Lemma 4

*I* has a solution if and only if $I[X\backslash S^I_{(P_{xv})}]$ has a solution.

### Proof

Again the “only if” implication is trivial so we focus on the other direction. Let us assume for the sake of contradiction that $I[X\backslash S^I_{(P_{xv})}]$ has a solution but *I* does not. In particular this implies that $I_{xv}$ does not have a solution, and then by Lemma 3 we know that $I_{xv}[X\backslash S^I_{(P_{xv})}]$ has no solution either. We define *Z* as a subset of $X\backslash S^I_{(P_{xv})}$ of minimum size such that $I_{xv}[Z]$ has no solution. Observe that $I_{xv}[Z]$ can only differ from *I*[*Z*] by having fewer values in the domain of the variables in $S_{(P_{xv})}$. Let $\phi$ be a solution to *I*[*Z*] such that $\phi (y) \in D_{xv}(y)$ for as many variables *y* as possible. Because $\phi$ is not a solution to $I_{xv}[Z]$, there exists $p_r \in Z \cap S_{(P_{xv})}$ and $p_j \in S^I_{(P_{xv})}$ such that $(p_j \rightarrow p_r) \in (P_{xv})$ and $\phi (p_r) \in \rho (p_j \rightarrow p_r)$ (recall that $\rho (p_j \rightarrow p_r)$ is the set of points removed by the AC algorithm in the domain of $p_r$ at step $(p_j \rightarrow p_r)$). By construction, $p_j \notin Z$.

First, let us assume that there exists a variable $y \in Z$, $y \ne p_r$ such that there is no $a_r \in D_{xv}(p_r)$ with $(\phi (y),a_r) \in R(y,p_r)$. This implies, in particular, that $\phi (y) \notin D_{xv}(y)$. We first prove that $R(y,p_r)$ and $R(p_j,p_r)$ are the only possible non-trivial constraints involving $p_r$ in $I_{xv}$. If there exists a fourth variable *z* such that $R(p_r,z)$ is non-trivial in $I_{xv}$, then there exist $a_r,b_r \in D_{xv}(p_r)$ and $a_z \in D_{xv}(z)$ such that $(a_r,a_z) \in R(p_r,z)$ but $(b_r,a_z) \notin R(p_r,z)$. By assumption we have $(\phi (y),a_r) \notin R(y,p_r)$ and $(\phi (y),\phi (p_r)) \in R(y,p_r)$. Finally, $b_r$ has a support $a_j \in D_{xv}(p_j)$ and $\phi (p_r) \in \rho (p_j \rightarrow p_r)$ so we have $(a_j,a_r) \in R(p_j,p_r)$ but $(a_j,\phi (p_r)) \notin R(p_j,p_r)$. This produces Q1 on $(p_r,y,p_j,z)$ with $p_r$ being the middle variable. Therefore, we know that $R(y,p_r)$ and $R(p_j,p_r)$ are the only possible non-trivial constraints involving $p_r$ in $I_{xv}$. However, in this case the variable $p_r$ has only one incident non-trivial constraint in $I_{xv}[Z]$, and hence $I_{xv}[Z]$ has a solution if and only if $I_{xv}[Z \backslash p_r]$ has one. This contradicts the minimality of *Z*, and for the rest of the proof we can assume that for every $y \in Z$ there exists some $a_r \ne \phi (p_r)$ such that $a_r \in D_{xv}(p_r)$ and $(\phi (y),a_r) \in R(y,p_r)$.

**Fig. 6:**
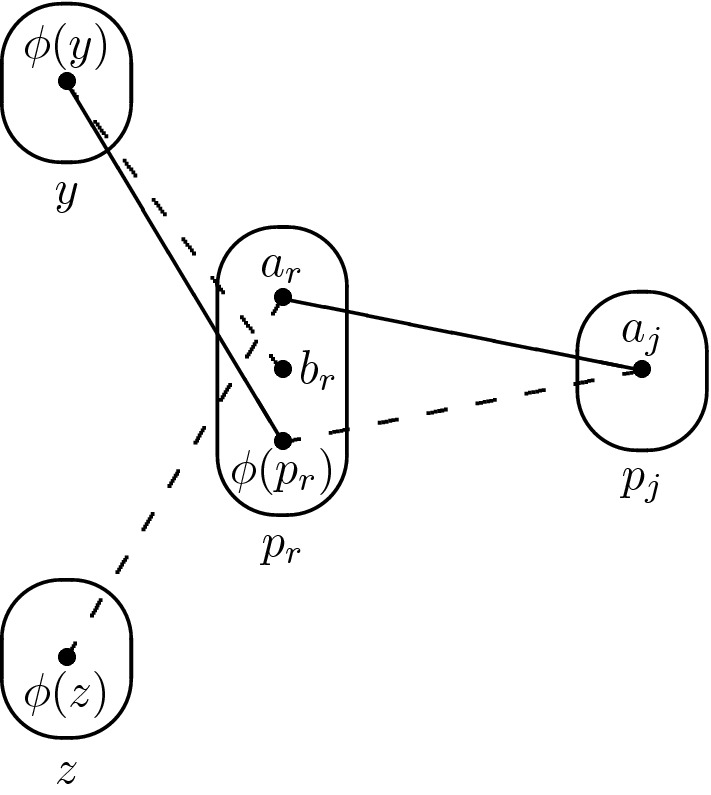
Some positive and negative edges between *y*, *z*, $p_j$ and $p_r$. The positive edges $\phi (y)a_r$ and $\phi (z)\phi (p_r)$ are omitted for clarity; $b_r$ is any value in $D_{xv}(p_r)$ that is not compatible with $\phi (y)$

Now, let $y \in Z$ be such that $y \ne p_r$ and $|\{ b \in D_{xv}(p_r) \mid (\phi (y),b) \in R(y,p_r) \}|$ is minimum. By the argument above, there exists $a_r \in D_{xv}(p_r)$ such that $(\phi (y),a_r) \in R(y,p_r)$ and $a_r \ne \phi (p_r)$. By the choice of $\phi$ and $p_r$, setting $\phi (p_r) = a_r$ would violate at least one constraint in *I*[*Z*], so there exists some variable $z \in Z$, $z \ne y$ such that $(\phi (z),a_r) \notin R(z,p_r)$. Furthermore, by arc consistency of $I_{xv}$ there exists $a_j \in D_{xv}(p_j)$ such that $(a_j,a_r) \in R(p_j,p_r)$. Recall that we picked $p_j$ in such a way that $\phi (p_r) \in \rho (p_j \rightarrow p_r)$, and so we have $(a_j,\phi (p_r)) \notin R(p_j,p_r)$. We summarize what we have in Fig. 6.

Observe that unless Q1 occurs, for every $b_r \in D_{xv}(p_r)$ such that $(\phi (y),b_r) \notin R(y,p_r)$ we also have $(\phi (z),b_r) \notin R(z,p_r)$. However, recall that $(\phi (y),a_r) \in R(y,p_r)$ so $\phi (z)$ is compatible with strictly fewer values in $D_{xv}(p_r)$ than $\phi (y)$. This contradicts the choice of *y*. It follows that setting $\phi (p_r) = a_r$ cannot violate any constraint in *I*[*Z*], which is impossible by our choice of $\phi$ - a final contradiction. $\square$

### Theorem 1

CSP($\overline{\hbox {Q1}}$) is solved by singleton arc consistency.

### Proof

Let $I \in$ CSP($\overline{\hbox {Q1}}$) be singleton arc consistent. Pick any variable *x* and value $v \in D(x)$. By singleton arc consistency the instance $I_{xv}$ does not have any empty domains. If $S^I_{(P_{xv})}$ is empty then *I* has a solution if and only if $I[X\backslash \{x\}]$ has one. Otherwise, by Lemma 4, *I* has a solution if and only if $I[X\backslash S^I_{(P_{xv})}]$ has one. In the latter case we must have $x \in S^I_{(P_{xv})}$, so overall we can conclude that *I* has a solution if and only if $I[X\backslash (S^I_{(P_{xv})}\cup \{x\})]$ has one. Because $I[X\backslash (S^I_{(P_{xv})}\cup \{x\})]$ is singleton arc consistent as well and $S^I_{(P_{xv})}\cup \{x\} \ne \emptyset$ we can repeat the procedure until $X\backslash (S^I_{(P_{xv})}\cup \{x\})$ is empty, at which point we may conclude that *I* has a solution. $\square$

## Tractability of R8 and R7-

Q1 and R8 (Fig. 2) are structurally dissimilar, but the idea of using $I_{xv}$ and the trace of the arc consistency algorithm to extract variables from *I* without altering satisfiability works in the case of R8 as well. We define a *star* to be a non-empty set of constraints whose scopes all intersect. The *centers* of a star are its variables of highest degree (every star with three or more variables has a unique center). The following lemma is the R8 analog of Lemma 1; the main differences are a slightly stronger prerequisite (no neighbourhood substitutable values) and that arc consistency leaves stars of non-trivial constraints instead of paths.

### Lemma 5

Let $I=(X,D,C) \in$ CSP($\overline{\hbox {R8}}$) be singleton arc consistent. Let $x \in X$, $v \in D(x)$ and consider the instance $I_{xv}$. After the removal of every neighbourhood substitutable value, every connected component of non-trivial constraints that intersect with $S_{(P_{xv})}$ is a star with a center in $S_{(P_{xv})}$.

### Proof

We proceed by induction. Suppose that all neighbourhood substitutable values have been removed. First, no connected component of non-trivial constraints may contain $p_0 = x$. Then, let $k \ge 0$ and suppose that every connected component of non-trivial constraints that intersect $\{p_i \mid i \le k \}$ is a star centered on $S_{(P_{xv})}$. Suppose also, for the sake of contradiction, that there exists a connected component $\mathcal {G}$ of non-trivial constraints that contains $p_{k+1}$ and that is *not* a star centered on $S_{(P_{xv})}$.

Let $p_j$, $j \le k$ be such that $(p_j \rightarrow p_{k+1}) \in (P_{xv})$. By the induction hypothesis, $p_j$ cannot be part of $\mathcal {G}$ and hence $R(p_j,p_{k+1})$ must be trivial. Furthermore, if every simple path of non-trivial constraints starting at $p_{k+1}$ had length 1 then $\mathcal {G}$ would be a star centered on $p_{k+1}$, which would contradict our assumption. Therefore, there exist two distinct variables $z_1,z_2 \notin \{p_j,p_{k+1}\}$ such that neither $R(p_{k+1},z_1)$ nor $R(z_1,z_2)$ is trivial (again, the claim $z_1,z_2 \ne p_j$ comes from the fact that $p_j$ is not part of $\mathcal {G}$).

Because $R(p_{k+1},z_1)$ is not trivial, there exist two distinct values $a_{k+1},b_{k+1} \in D_{xv}(p_{k+1})$ and $a_1 \in D_{xv}(z_1)$ such that $(a_{k+1},a_1) \in R(p_{k+1},z_1)$ but $(b_{k+1},a_1) \notin R(p_{k+1},z_1)$. Furthermore, $R(p_j,p_{k+1})$ is trivial and hence there exists $a_j \in D_{xv}(p_j)$ such that $(a_j,a_{k+1}),(a_j,b_{k+1}) \in R(p_j,p_{k+1})$. Finally, since $(p_j \rightarrow p_{k+1}) \in (P_{xv})$ some propagation must have taken place in the domain of $p_{k+1}$, and hence there exists $c_{k+1}$ such that $(a_j,c_{k+1}) \notin R(p_j,p_{k+1})$. We can summarize what we have in the following picture (the tuple $(a_1,a_2)$ comes from the fact that $a_1$ must have a support in $R(z_1,z_2)$). 
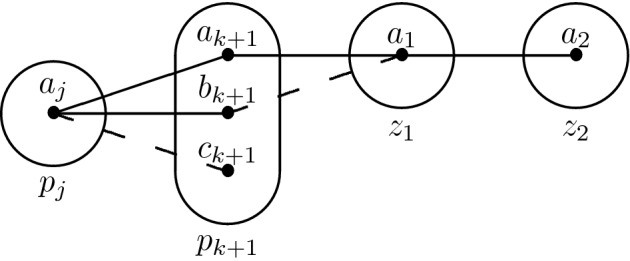


*First Case: There Exists*  $\varvec{b_1 \in D_{xv}(z_1)}$ *Such That*  $\varvec{(b_1,a_2) \notin R(z_1,z_2)}$ For R8 not to occur, $(b_{k+1},b_1)$ must not belong to $R(p_{k+1},z_1)$. By arc consistency, $b_1$ must be connected to some $d_{k+1} \in D_{xv}(p_{k+1})$. If there exists one such $d_{k+1}$ such that $(d_{k+1},a_1) \notin R(p_{k+1},z_1)$, then R8 occurs again, so $a_1$ dominates $b_1$ in the constraint $R(p_{k+1},z_1)$. However, recall that all neighbourhood substitutable values have been removed, so there must exist a variable $z_3$ (potentially equal to $z_2$, but different from $p_j,p_{k+1},z_1$) and $a_3 \in D_{xv}(z_3)$ such that $(b_1,a_3) \in R(z_1,z_3)$ but $(a_1,a_3) \notin R(z_1,z_3)$. Finally, because $b_{k+1}$ is arc consistent, there exists $c_1 \in D(z_1)$ such that $(b_{k+1},c_1) \in R(p_{k+1},z_1)$. We obtain the following two structures, which may only differ on the last constraint. 
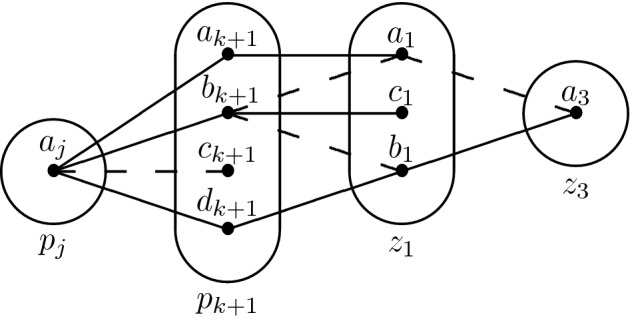

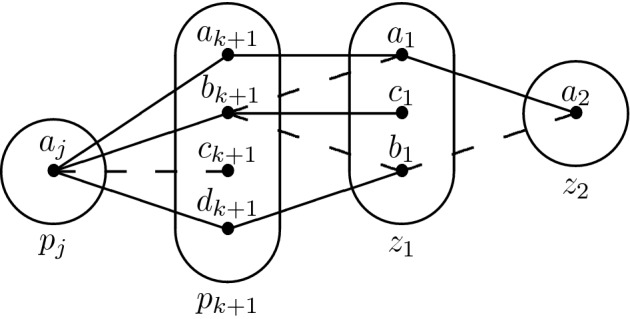


The key observation here is that whenever $a_1$ or $b_1$ is compatible with any value *v* of a fourth variable $y \notin \{p_j, p_{k+1}, z_1\}$, then $c_1$ is compatible with *v* as well unless R8 occurs. Thus, the only constraint on which $c_1$ may not dominate both $a_1$ and $b_1$ is $R(p_{k+1},z_1)$. However, if $(d_{k+1},c_1) \notin R(p_{k+1},z_1)$ then R8 occurs in $(p_j,p_{k+1},z_1,z_2)$, and if $(a_{k+1},c_1) \notin R(p_{k+1},z_1)$ then R8 occurs in $(p_j,p_{k+1},z_1,z_3)$; this is true for any choice of $a_{k+1}$ and $d_{k+1}$ so $c_1$ dominates both $a_1$ and $b_1$ in $R(p_{k+1},z_1)$ - a contradiction, since it means that $a_1$ and $b_1$ should have been removed by neighbourhood substitution.

*Second Case: There Does not Exist* $\varvec{b_1 \in D_{xv}(z_1)}$  *Such That* $\varvec{(b_1,a_2) \notin R(z_1,z_2)}$ *for any Choice of*  $\varvec{z_2}$ This means that we must have $(v_1,v_2) \in R(z_1,z_2)$ for all $v_1 \in D_{xv}(z_1)$ and $v_2 \in D_{xv}(z_2)$ such that $(a_1,v_2) \in R(z_1,z_2)$. Putting this together with the fact that by hypothesis $R(z_1,z_2)$ is not trivial, there exists $b_2 \in D_{xv}(z_2)$ such that $(a_1,b_2) \notin R(z_1,z_2)$. Then $b_2$ must have a support $(b_1,b_2)$ in $R(z_1,z_2)$, and $b_1$ must have a support $(d_{k+1},b_1)$ in $R(p_{k+1},z_1)$. Because $R(p_j,p_{k+1})$ is trivial, $(a_j,d_{k+1}) \in R(p_j,p_{k+1})$. Let us update our picture: 
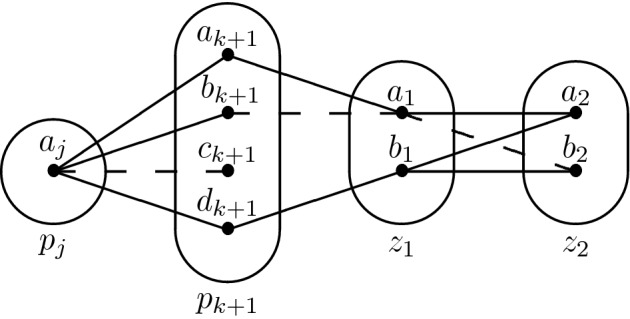


Observe that $a_{k+1}$ is an arbitrary value of $D_{xv}(p_{k+1})$ that is compatible with $a_1$. If $(a_{k+1},b_1) \notin R(p_{k+1},z_1)$, then R8 occurs. Hence, every value compatible with $a_1$ in *every* constraint involving $z_1$ is also compatible with $b_1$. This means that $a_1$ should have been removed by neighbourhood substitution - a final contradiction. $\square$

In the proof of SAC-solvability of Q1, only inner variables are extracted from the instance. The above lemma suggests that in the case of R8 it is more convenient to extract all variables in $S_{(P_{xv})}$, plus any variable that can be reached from those via a non-trivial constraint.

### Lemma 6

Let $I=(X,D,C) \in$ CSP($\overline{\hbox {R8}}$) be singleton arc consistent. Let $x \in X$, $v \in D(x)$ and consider the instance $I_{xv}$. After the removal of every neighbourhood substitutable value, there exists a partition $(X_1,X_2)$ of $X$ such that

- $$S_{(P_{xv})}\subseteq X_1$$;
- $$\forall (x,y) \in X_1 \times X_2$$, *R*(*x*, *y*) is trivial;
- Every connected component of non-trivial constraints with scopes subsets of $X_1$ is a star.

### Proof

Let $X_1 = S_{(P_{xv})}\cup \{ \, z \in X\, \mid \, \exists y \in S_{(P_{xv})}\, : \, D_{xv}(y) \times D_{xv}(z) \not \subseteq R(y,z) \, \}$. We have $S_{(P_{xv})}\subseteq X_1$, and by construction every non-trivial constraint between $y \in X_1$ and $z \notin X_1$ must be such that $y \notin S_{(P_{xv})}$ and *y* is adjacent to a variable in $S_{(P_{xv})}$ via a non-trivial constraint. By Lemma 5 this is impossible, and hence there is no non-trivial constraint between $X_1$ and $X\backslash X_1$. The last property is immediate by Lemma 5. $\square$

### Theorem 2

CSP($\overline{\hbox {R8}}$) is solved by singleton arc consistency.

### Proof

Let $I \in$ CSP($\overline{\hbox {R8}}$) and suppose that *I* is singleton arc consistent. Let $x \in X$ and $v \in D(x)$. Because of singleton arc consistency the instance $I_{xv}$ has no empty domains. We remove all neighbourhood substitutable values from $I_{xv}$. By Lemma 6, the variable set of $I_{xv}$ can be divided into two parts $X_1,X_2$ such that $I_{xv}$ has a solution if and only if both $I_{xv}[X_1]$ and $I_{xv}[X_2]$ are satisfiable. $I_{xv}[X_1]$ is an arc consistent instance with no cycle of non-trivial constraints, and hence is satisfiable. $I_{xv}[X_2]$ is exactly $I[X_2]$ with some neighbourhood substitutable values removed because no variable in $X_2$ was affected by propagation after *x* was assigned. Call this new instance $I[X_2]'$. Because $I[X_2]'$ is singleton arc consistent as well (being singleton arc consistent is invariant under projection and removal of neighbourhood substitutable values), we can repeat the same reasoning on $I[X_2]'$. At each step the set $X_1$ cannot be empty (it contains *x*) so this procedure will always terminate, and because each $I[X_1]$ has a solution *I* has a solution as well. $\square$

Our proof of the SAC-solvability of R7- (Fig. 3) follows a similar reasoning, with two main differences. First, branching on just any variable-value pair (as we did for Q1 and R8) may lead to a subproblem that is *not* solved by arc consistency. However, once the right assignment is made the reward is much greater as all constraints involving a variable whose domain has been reduced by arc consistency must become trivial *except at most one*.

**Fig. 7:**
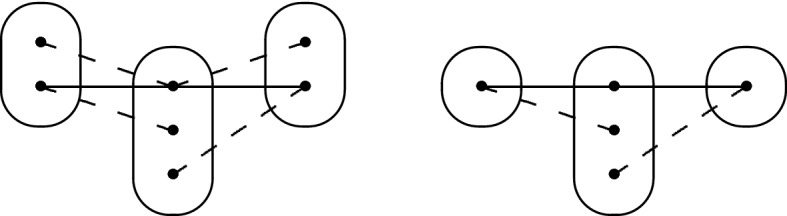
The patterns ${\hat{\hbox {M}}}$ (left) and $\hbox {V}_{2}$ (right)

Finding out which variable-value pair (*x*, *v*) we should branch on is tricky. We first show that the above property is guaranteed to hold if (*x*, *v*) is the meet point of the positive edges in a particular pattern ${\hat{\hbox {M}}}$ (Fig. 7). However, ${\hat{\hbox {M}}}$ is an NP-hard pattern [17] so it might happen that ${\hat{\hbox {M}}}$ does not occur at all in the instance. To handle this problem we define a weaker pattern $\hbox {V}_{2}$ (Fig. 7), whose absence is known to imply SAC-solvability (because it is a sub-pattern of R8) and hence can be safely assumed to occur somewhere. Our strategy is to branch on the assignment that corresponds to the meet point of the positive edges in $\hbox {V}_{2}$ and attempt to prove that the above property holds by induction, following the trace of the AC algorithm. We then show that if the induction started from V$_2$ breaks then ${\hat{\hbox {M}}}$ must occur somewhere - a win-win situation.

### Lemma 7

Let $I=(X,D,C) \in$ CSP($\overline{\hbox {R7-}}$) be singleton arc consistent. Let $x \in X$ be such that ${\hat{\hbox {M}}}$ occurs on (*y*, *x*, *z*) with *x* the middle variable and *v* be the value in *D*(*x*) that is the meet point of the two positive edges. Then every constraint whose scope contains a variable in $S_{(P_{xv})}$ is trivial in $I_{xv}$, except possibly *R*(*y*, *z*).

### Proof

We prove the claim by induction. Every constraint with $p_0 = x$ in its scope is trivial. Let $k \ge 0$ and suppose that the claim holds for every constraint whose scope contains a variable in $\{p_i \mid i \le k\}$. Let $w \in X\backslash \{p_i \mid i \le k\}$ be a variable such that $R(p_{k+1},w)$ is not trivial in $I_{xv}$ and $\{p_{k+1},w\} \ne \{y,z\}$. Let $p_j$ be such that $(p_j \rightarrow p_{k+1}) \in (P_{xv})$, with $j \le k$. Because $R(p_{k+1},w)$ is not trivial and $I_{xv}$ is arc consistent, there exist $a_{k+1},b_{k+1} \in D_{xv}(p_{k+1})$ and $a_w \in D_{xv}(w)$ such that $(a_{k+1},a_w) \in R(p_{k+1},w)$ and $(b_{k+1},a_w) \notin R(p_{k+1},w)$.

If $R(p_j,p_{k+1})$ is trivial, then there exists $a_j \in D_{xv}(p_j)$ such that $(a_j,a_{k+1}),(a_j,b_{k+1}) \in R(p_j,p_{k+1})$ and $c_{k+1}$ such that $(a_j,c_{k+1}) \notin R(p_j,p_{k+1})$ ($c_{k+1}$ is one of the values that were eliminated by arc consistency at step $(p_j \rightarrow p_{k+1})$). Then, if $p_j \ne x$ there exists $p_i$, $i < j$ such that $(p_i \rightarrow p_j) \in (P_{xv})$. By arc consistency and because some propagation must have taken place in the domain of $p_j$ at step $(p_i \rightarrow p_j)$, there exists $a_i \in D_{xv}(p_i)$ and $b_j$ such that $(a_i,a_j) \in R(p_i,p_j)$ and $(a_i,b_j) \notin R(p_i,p_j)$. It follows that R7- occurs on $(p_i,p_j,p_{k+1},w)$, a contradiction. On the other hand, if $p_j = x$ then we obtain the same contradiction by using either *y* or *z* (the one which does not appear in $\{p_{k+1},w\}$) instead of $p_i$.

By the induction hypothesis, if $R(p_j,p_{k+1})$ is not trivial then $\{p_j,p_{k+1}\} = \{y,z\}$. By symmetry we can assume $p_{k+1} = z$. *R*(*x*, *z*) is trivial, so $\{ (v,a_{k+1}), (v,b_{k+1}) \} \subseteq R(x,z)$. Furthermore, ${\hat{\hbox {M}}}$ occurs on (*y*, *x*, *z*) so there exist $c_{k+1}$ such that $(v,c_{k+1}) \notin R(x,z)$ and $a_y,b_x$ such that $(a_y,v) \in R(y,z)$ but $(a_y,b_x) \notin R(y,z)$. Then, R7- occurs on (*y*, *x*, *z*, *w*), a contradiction.

In both cases the induction holds, so the claim follows. $\square$

### Lemma 8

Let $I=(X,D,C) \in \hbox {CSP} (\overline{\hat{\hbox {M}}}) \cap$ CSP($\overline{\hbox {R7-}}$) be singleton arc consistent. Let $x \in X$ be such that V$_2$ occurs on (*y*, *x*, *z*) with *x* the middle variable and *v* be the value in *D*(*x*) that is the meet point of the two positive edges. Then every constraint whose scope contains a variable in $S_{(P_{xv})}$ is trivial in $I_{xv}$, except possibly *R*(*y*, *z*).

### Proof

The proof follows the same idea as for Lemma 7. However, in this case the fact that ${\hat{\hbox {M}}}$ does not occur is critical in order to keep the induction going.

Again, every constraint with $p_0 = x$ in its scope is trivial. Let $k \ge 0$ and suppose that the claim holds for every constraint whose scope contains a variable in $\{p_i \mid i \le k\}$. Let $w \in X\backslash \{p_i \mid i \le k\}$ be a variable such that $R(p_{k+1},w) \in C$ is not trivial in $I_{xv}$ and $\{p_{k+1},w\} \ne \{y,z\}$. Let $p_j$ be such that $(p_j \rightarrow p_{k+1}) \in (P_{xv})$. Because $R(p_{k+1},w)$ is not trivial and $I_{xv}$ is arc consistent, there exist $a_{k+1},b_{k+1} \in D_{xv}(p_{k+1})$ and $a_w \in D_{xv}(w)$ such that $(a_{k+1},a_w) \in R(p_{k+1},w)$ and $(b_{k+1},a_w) \notin R(p_{k+1},w)$.

If $R(p_j,p_{k+1})$ is trivial we can proceed exactly as in the proof of Lemma 7, so let us focus on the case where $R(p_j,p_{k+1})$ is not trivial. By induction we must have $\{p_j,p_{k+1}\} = \{y,z\}$. We assume without loss of generality that $p_{k+1} = z$. If $(x \rightarrow z) \in (P_{xv})$ then we can use *x* instead of *y* to bring us to the case where $R(p_j,p_{k+1})$ is trivial, so let us assume $(x \rightarrow z) \notin (P_{xv})$. Then, if $(x \rightarrow y) \notin (P_{xv})$ there exists $p_i,p_l$ such that $i,l \le k$, $(p_i \rightarrow y) \in (P_{xv})$ and $(p_l \rightarrow p_i) \in (P_{xv})$. However, by induction $R(p_i,y)$ is trivial and thus *R*(*y*, *z*) should have been trivial as well (otherwise, the argument of Lemma 7 produces R7- on $(p_l,p_i,y,z)$). We can therefore assume that $(x \rightarrow y) \in (P_{xv})$ to work our way towards a contradiction. In particular, this means that there exists $c_y$ such that $(v,c_y) \notin R(x,y)$ ($c_y$ being a value eliminated by arc consistency). Because $\hbox {V}_{2}$ occurs on (*y*, *x*, *z*), there exists $a_y \in D_{xv}(y)$ and $a_x$ such that $(v,a_y) \in R(x,y)$ and $(a_x,a_y) \notin R(x,y)$. The picture below summarises the structure derived from the arguments above. Observe that we can always assume that either $(a_y,a_{k+1}) \in R(y,z)$ or $(a_y,b_{k+1}) \in R(y,z)$ by replacing $a_{k+1}$ or $b_{k+1}$ with a support for $a_y$ in *R*(*y*, *z*). 
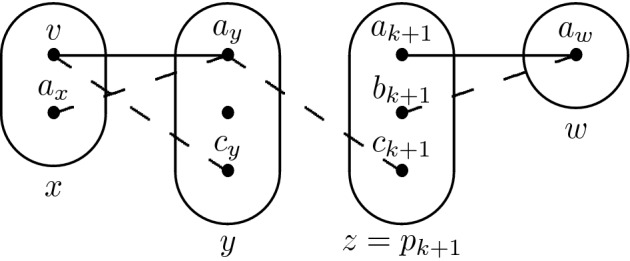


If $$(a_y,a_{k+1}) \in R(y,z)$$, then unless R7- occurs on (*x*, *y*, *z*, *w*) we must have $$(a_y,b_{k+1}) \notin R(y,z)$$. By arc consistency of $$I_{xv}$$, there exists $$b_y \in D_{xv}(y)$$ such that $$(b_y,b_{k+1}) \in R(y,z)$$, $$(b_y,c_{k+1}) \notin R(y,z)$$ (since $$c_{k+1}$$ was eliminated by arc consistency) and because *R*(*x*, *y*) is trivial we have $$(v,b_y) \in R(x,y)$$. Again, unless R7- occurs on (*x*, *y*, *z*, *w*) we have $$(b_y,a_{k+1}) \notin R(y,z)$$. At this point one can observe in the picture below that the pattern $${\hat{\hbox {M}}}$$ occurs on (*x*, *y*, *z*) with the meet point of the two solid lines being $$a_y$$. This contradicts the assumption that $$I \in $$$$\hbox {CSP}(\overline{\hat{\hbox {M}}})$$. 
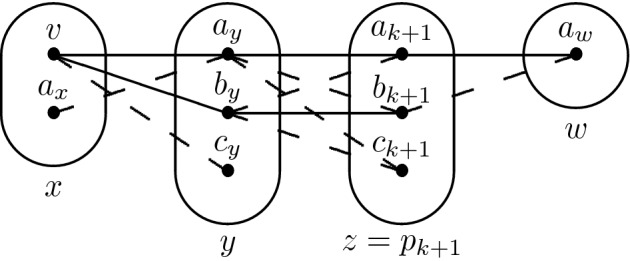


The case where $(a_y,b_{k+1}) \in R(y,z)$ is almost symmetric. Because R7- does not occur, we must have $(a_y,a_{k+1}) \notin R(y,z)$. By arc consistency, there exists some $b_y \in D_{xv}(y)$ such that $(b_y,a_{k+1}) \in R(y,z)$, $(b_y,c_{k+1}) \notin R(y,z)$ and because *R*(*x*, *y*) is trivial we have $(v,b_y) \in R(x,y)$. It follows from the absence of R7- that $(b_y,b_{k+1}) \notin R(y,z)$, which create the pattern ${\hat{\hbox {M}}}$ on (*x*, *y*, *z*) with its meet point being $a_y$, as shown in the picture below. 
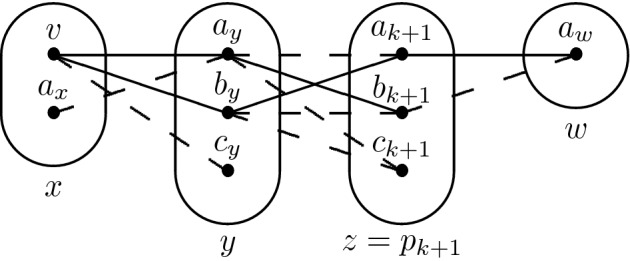


This final contradiction completes the proof. $\square$

### Theorem 3

CSP($\overline{\hbox {R7-}}$) is solved by singleton arc consistency.

### Proof

Let $I = (X,D,C) \in$ CSP($\overline{\hbox {R7-}}$), and suppose that enforcing SAC did not lead to a wipeout of any variable domain. If $\hbox {V}_{2}$ does not occur in *I* then it has a solution (recall that absence of $\hbox {V}_{2}$ ensures solvability by SAC), so let us assume that $\hbox {V}_{2}$ occurs. Let $x \in X$ and $v \in D(x)$ be such that *v* is the meet point of solid edges of ${\hat{\hbox {M}}}$ if ${\hat{\hbox {M}}}$ occurs in *I*, and the meet point of V$_2$ otherwise. *I* is SAC so the instance $I_{xv}$ has no empty domains. By Lemmas 7 and 8, there is at most one non-trivial constraint in $I_{xv}[S_{(P_{xv})}]$ so by arc consistency for every $x_1 \in S_{(P_{xv})}$ and $v_1 \in D_{xv}(x_1)$ there is a solution $\phi$ to $I_{xv}[S_{(P_{xv})}]$ such that $\phi (x_1) = v_1$. Furthermore, $I_{xv}[X\backslash S_{(P_{xv})}] = I[X\backslash S_{(P_{xv})}]$ and there is at most one non-trivial constraint in $I_{xv}$ with one endpoint in $S_{(P_{xv})}$ and the other in $X\backslash S_{(P_{xv})}$. By combining the two properties we obtain that $I_{xv}$ has a solution if and only if $I[X\backslash S_{(P_{xv})}]$ has one. Because $I[X\backslash S_{(P_{xv})}]$ is SAC and R7- still does not occur, we can repeat the operation until we have a solution to the whole instance. $\square$

## Tractability of Q2 and R5

For our last two proofs of SAC-decidability, we depart from the trace technique. Our fundamental goal, however, remains the same: find an operation which shrinks the instance without altering satisfiability, introducing the pattern or losing singleton arc consistency. For Q2 this operation is BTP-merging [19] and for R5 it is removing constraints.

**Fig. 8:**
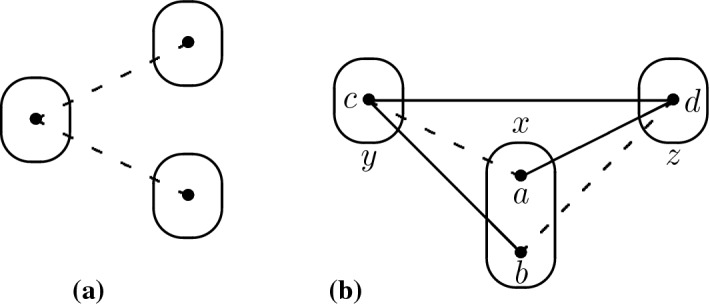
**a** The pattern V$^{-}$ and **b** the associated broken-triangle pattern (BTP)

Consider the pattern V$^{-}$ shown in Fig. 8a. We say that V$^{-}$ occurs at point *a* or at variable *x* if $a \in D(x)$ is the central point of the pattern in the instance. The pattern V$^{-}$ is known to be tractable since all instances in $\hbox {CSP}(\overline{\hbox {V}^{-}})$ satisfy the joint-winner property [22]. However, we show a slightly different result, namely that singleton arc consistency is sufficient to solve instances in which V$^{-}$ only occurs at degree-2 variables.

### Lemma 9

Instances in which V$^{-}$ only occurs at degree-2 variables are solved by singleton arc consistency.

### Proof

Singleton arc consistency only eliminates values from domains and thus cannot increase the degree of a variable nor introduce the pattern V$^{-}$. Hence, singleton arc consistency cannot lead to the occurrence of the pattern V$^{-}$ at a variable of degree greater than two. Therefore it is sufficient to show that any SAC instance *I* in which V$^{-}$ only occurs at degree-2 variables is satisfiable.

We will show that it is always possible to find an independent partial solution, i.e. an assignment to a non-empty subset of the variables of *I* which is compatible with all possible assignments to the other variables. A solution can be found by repeatedly finding independent partial solutions. If *I* has only degree-2 variables, then it is folklore (and easy to show) that singleton arc consistency implies satisfiability. So we only need to consider the case in which *I* has at least one variable $x_1$ of degree greater than or equal to three. Choose an arbitrary value $a_1 \in D(x_1)$. If this assignment is compatible with all assignments to all other variables, then this is the required independent partial solution, so suppose that there is a negative edge $a_1b$ where $b \in D(x_2)$ for some variable $x_2$. By assumption, since $x_1$ has degree greater than or equal to three, the pattern V$^{-}$ does not occur at $x_1$ and hence the assignment $(x_1,a_1)$ is compatible with all assignments to all variables other than $x_1,x_2$.

**Fig. 9:**
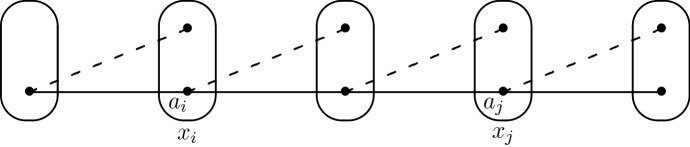
The edge $a_ia_j$ must be positive, otherwise the pattern V$^{-}$ would occur at $a_i$ and variable $x_i$ would have degree at least three. In the special case $i=1$, this follows from our choice of $x_1$ to be a variable of degree at least three

Now suppose that we have a partial assignment $(x_1,a_1),\ldots ,(x_k,a_k)$, as shown in Fig. 9, such that

1. for $$i=1,\ldots ,k$$, $$a_i \in D(x_i)$$,
2. for $i=1,\ldots ,k-1$, $\exists b \in D(x_{i+1})$ such that $a_ib$ is a negative edge.
3. for $i=1,\ldots ,k-1$, $a_ia_{i+1}$ is a positive edge,

The assignments $(x_i,a_i)$ ($i=1,\ldots ,k$) are all compatible with each other, otherwise the pattern V$^{-}$ would occur at a variable of degree three or greater, as illustrated in Fig. 9. Furthermore, for the same reason, the assignments $(x_i,a_i)$ ($i=1,\ldots ,k-1$) are all compatible with all possible assignments to all variables other than $x_1,\ldots ,x_k$. It only remains to consider the compatibility of $a_k$ with the assignments to variables other than $x_1,\ldots ,x_k$.

If the assignment $(x_k,a_k)$ is compatible with all assignments to all variables other than $x_1,\ldots ,x_k$, then we have an independent partial solution to $x_1,\ldots ,x_k$. On the other hand, if for some $x_{k+1} \notin \{x_1,\ldots ,x_k\}$, there is $b \in D(x_{k+1})$ such that $a_kb$ is a negative edge, then by (singleton) arc consistency there exists $a_{k+1} \in D(x_{k+1})$ such that $a_ka_{k+1}$ is a positive edge and we have a larger partial assignment with the above three properties. Therefore, we can always add another assignment until the resulting partial assignment is an independent partial solution (or we have assigned all variables). $\square$

Two values $a,b \in D(x)$ are *BTP-mergeable* [19] if there are not two other distinct variables $y,z \ne x$ such that there exist $c \in D(y)$ and $d \in D(z)$ with *ad*, *bc*, *cd* positive edges and *ac*, *bd* negative edges as shown in Fig. 8b. The *BTP-merging* operation consists in merging two BTP-mergeable points $a,b \in D(x)$: the points *a*, *b* are replaced by a new point *c* in *D*(*x*) such that for all other variables $w \ne x$ and for all $d \in D(w)$, *cd* is a positive edge if at least one of *ad*, *bd* was a positive edge (a negative edge otherwise). BTP-merging preserves satisfiability [19].

### Lemma 10

Let *P* be a pattern in which no point occurs in more than one positive edge. Then the BTP-merging operation cannot introduce the pattern *P* in an instance $I \in$ CSP($\overline{\hbox {P}}$).

### Proof

Suppose that the pattern *P* occurs in an instance $I'$ obtained by BTP-merging of two points *a*, *b* in *I* to create a new point *c* in $I'$. From the assumptions about *P*, we know that *c* belongs to any number of negative edges $ce_1,\ldots ,ce_r$, but at most one positive edge *cd* in the occurrence of *P* in $I'$. By the definition of merging, in *I* one of *ad*, *bd* must have been a positive edge and all of $ae_1,\ldots ,ae_r$ and $be_1,\ldots ,be_r$ must have been negative. Without loss of generality, suppose that *ad* was a positive edge. But then the pattern *P* occurred in *I* (on *a* instead of *c*) which is a contradiction. $\square$

Since Q2 has no point which occurs in more than one positive edge, we can deduce from Lemma 10 that Q2 cannot be introduced by BTP-merging. We then combine this property with Lemma 9 by proving that V$^-$ can only occur at degree-2 variables in any instance of CSP($\overline{\hbox {Q2}}$) with no BTP-mergeable values.

### Theorem 4

CSP($\overline{\hbox {Q2}}$) is solved by singleton arc consistency.

### Proof

Let $I \in$ CSP($\overline{\hbox {Q2}}$). Since establishing singleton arc consistency cannot introduce patterns, and hence in particular cannot introduce Q2, we can assume that *I* is SAC. Let $I'$ be the result of applying BTP-merging operations to *I* until convergence. By Lemma 10, we know that $I' \in$ CSP($\overline{\hbox {Q2}}$). Furthermore, since BTP-merging only weakens constraints (in the sense that the new value *c* is constrained less than either of the values *a*, *b* it replaces), it cannot destroy singleton arc consistency; hence $I'$ is SAC. By Lemma 9, it suffices to show that V$^{-}$ cannot occur in $I'$ at variables of degree three or greater.

**Fig. 10:**
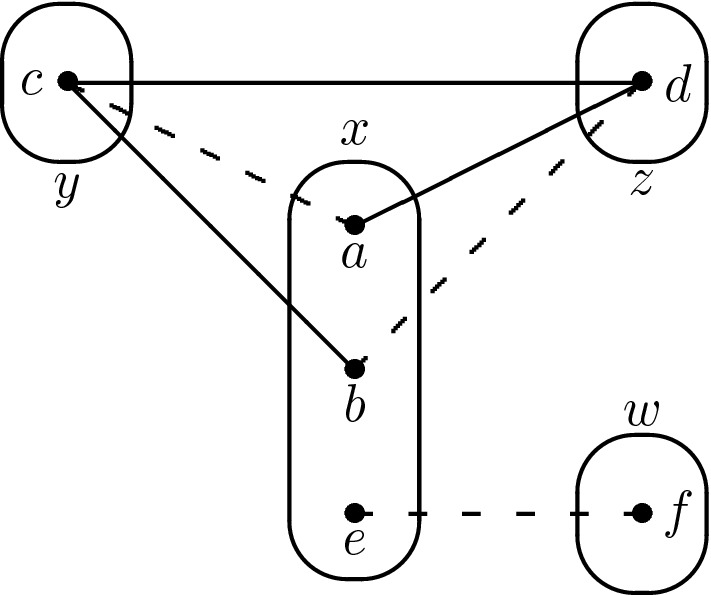
The pattern V$^{-}$ cannot occur at *a* if Q2 does not occur in the instance

Consider an arbitrary point $a \in D(x)$ at a variable *x* which is of degree three or greater. We will show that V$^{-}$ cannot occur at *a*, which will complete the proof. If *a* belongs to no negative edge then clearly V$^{-}$ cannot occur at *a*. The existence of a negative edge and the (singleton) arc consistency of *I* implies that there there is some other value $b \in D(x)$. Since *a*, *b* cannot be BTP-merged, there must be other variables *y*, *z* and values $c \in D(y)$, $d \in D(z)$ with *ad*, *bc*, *cd* positive edges and *ac*, *bd* negative edges, as shown in Fig. 10. Now since Q2 does not occur in *I*, we can deduce that *a* and *b* are connected by positive edges to all points in *D*(*v*) for $v \notin \{x,y,z\}$. Since *x* is of degree three or greater, there must therefore be another point $e \in D(x) \setminus \{a,b\}$ and a negative edge *ef* where $f \in D(w)$ for some $w \notin \{x,y,z\}$ (as shown in Fig. 10). By applying the same argument as above, knowing that *a*, *e* cannot be BTP-merged, we can deduce that *a* and *e* are connected by positive edges to all points in *D*(*v*) for $v \notin \{x,y,w\}$. Hence, *a* can only be connected by negative edges to points in *D*(*y*). It follows that the pattern V$^{-}$ cannot occur at *a*, which completes the proof. $\square$

That only leaves R5. Removing constraints cannot introduce R5 because it is a monotone pattern, so we can apply repeatedly the following lemma to obtain our last result.

**Fig. 11:**
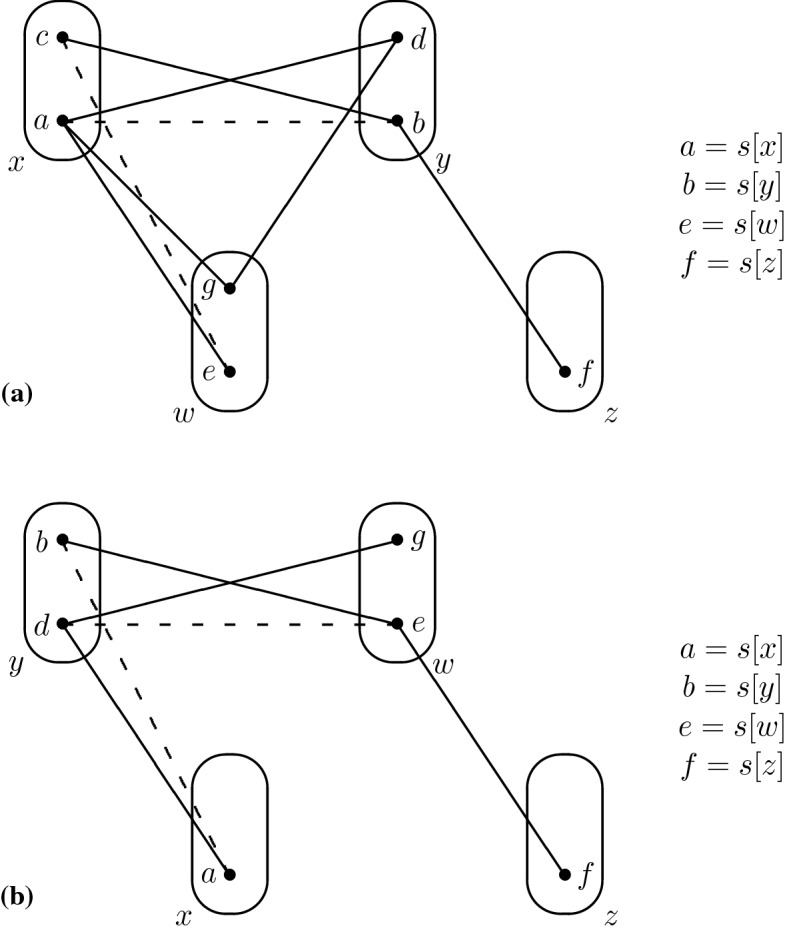
Since the pattern R5 does not occur in *I*, we can deduce that **a***df* is a positive edge, and **b***gf* is a positive edge

### Lemma 11

If the pattern R5 does not occur in a singleton arc consistent binary CSP instance *I*, then removing any constraint leaves the satisfiability of *I* invariant.

### Proof

Suppose that the pattern R5 does not occur in the instance *I* and that *I* is singleton arc consistent. Let $I'$ be the instance which results when we eliminate the constraint between an arbitrary pair of variables *x* and *y*. Suppose that *s* is a solution to $I'$. It suffices to exhibit a solution to *I*. We use *s*[*z*] to denote the value assigned to variable *z* in *s*. Let $a=s[x]$ and $b=s[y]$. If *ab* is a positive edge in *I* than *s* is also a solution to *I*, so we assume that *ab* is a negative edge.

By (singleton) arc consistency, there exists $c \in D(x)$ such that *bc* is a positive edge. Either we can replace *a* in *s* by *c* to produce a solution to *I*, or there is some variable $w \notin \{x,y\}$ such that *ce* is a negative edge where $e = s[w]$. By singleton arc consistency, there exist $d \in D(y)$ and $g \in D(w)$ such that *ad*, *ag* and *dg* are positive edges. Consider any variable $z \notin \{x,y,w\}$. We have the situation in *I* shown in Fig. 11a where $f = s[z]$. The positive edges *ae* and *bf* follow from the fact that *s* is a solution to $I'$. Now, since R5 does not occur in *I*, we can deduce that *df* is a positive edge. Recall that the variable *z* was any variable other than *x*, *y* or *w*.

Since $d \in D(y)$ is compatible with $a=s[x]$, we have just shown that *d* can only be incompatible with *s*[*z*] when $z=w$. Thus, either we can replace *b* by *d* to produce a solution to *I*, or *de* is a negative edge. In this latter case, consider any variable $z \notin \{x,y,w\}$ and again denote *s*[*z*] by *f*. We have the situation in *I* shown in Fig. 11b. The positive edges *be* and *ef* follow from the fact that *s* is a solution to $I'$. Since the pattern R5 does not occur in *I*, we can deduce that *gf* is a positive edge. But then we can replace *b* by *d* and *e* by *g* in *s* to produce a solution to *I*. $\square$

Note that Lemma 11 is technically true for all SAC-solvable patterns (not only R5); this is simply the only case where we are able to prove it directly.

### Theorem 5

CSP($\overline{\hbox {R5}}$) is solved by singleton arc consistency.

### Proof

Establishing singleton arc consistency preserves satisfiability and cannot introduce any pattern and, hence in particular, cannot introduce R5. Consider a SAC instance $I \in$ CSP($\overline{\hbox {R5}}$) which has non-empty domains. By Lemma 11, we can eliminate any constraint. The resulting instance is still SAC. Furthermore, R5 has not been introduced since R5 is monotone. Therefore, we can keep on eliminating constraints until all constraints have been eliminated. The resulting instance is trivially satisfiable and hence so was the original instance *I*. It follows that singleton arc consistency decides all instances in CSP($\overline{\hbox {R5}}$). $\square$

## A Necessary Condition for Solvability by SAC

**Fig. 12:**
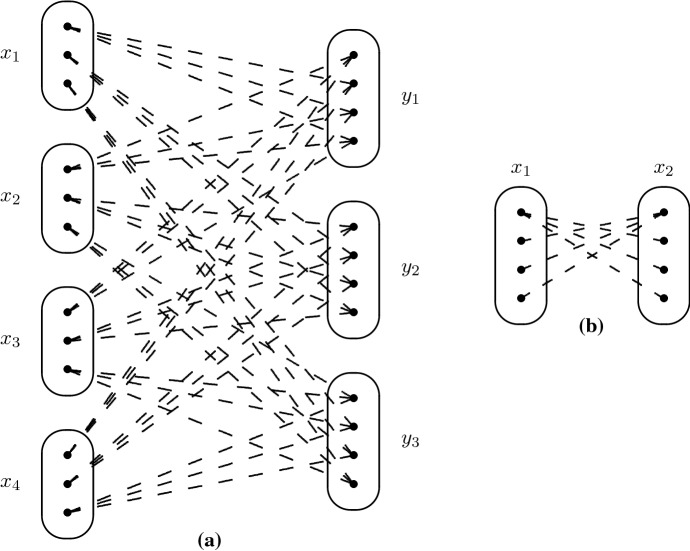
**a** The instance $I_{3,4}$ which is SAC but has no solution. **b** The constraint between variables $x_1$ and $x_2$ in instance $I_5$

In order to establish some basic properties of patterns solvable by SAC, we first show that several small patterns are not solvable by SAC. In order to do this, we consider the following instances:

- $I_{4}^{3COL}$: corresponds to 3-colouring the complete graph on 4 vertices, i.e. four variables $x_1,\ldots ,x_4$ with domains $D(x_i) = \{1,2,3\}$ ($i=1,\ldots ,4$) and the six inequality constraints: $x_i \ne x_j$ ($1 \le i < j \le 4$).
- $I_{3,4}$: corresponds to an alternative encoding of 3-colouring the complete graph on 4 vertices: three new variables $y_1,y_2,y_3$ are introduced such that $y_j=i$ if variable $x_i$ is assigned colour *j*. There are now seven variables ($x_1$, $x_2$, $x_3$, $x_4$, $y_1$, $y_2$, $y_3$) with domains $D(x_i) = \{1,2,3\}$ ($i=1,2,3,4$), $D(y_i) = \{1,2,3,4\}$ ($i=1,2,3$) and constraints $(x_i = j) \Rightarrow (y_j = i)$ ($i=1,2,3,4$; $j=1,2,3$). $I_{3,4}$ is shown in Fig. 12a (in which only negative edges are shown so as not to clutter up the figure).
- $$I_5$$: five variables ($$x_1,\ldots ,x_5$$) each with domain $$\{1,2,3,4\}$$ and the constraints $$(x_i = j-1)$$$$\Leftrightarrow $$$$(x_j=i)$$ for all *i*, *j* such that $$1 \le i < j \le 5$$. One constraint of this instance is shown in Fig. 12b (again only negative edges are shown).

**Fig. 13:**
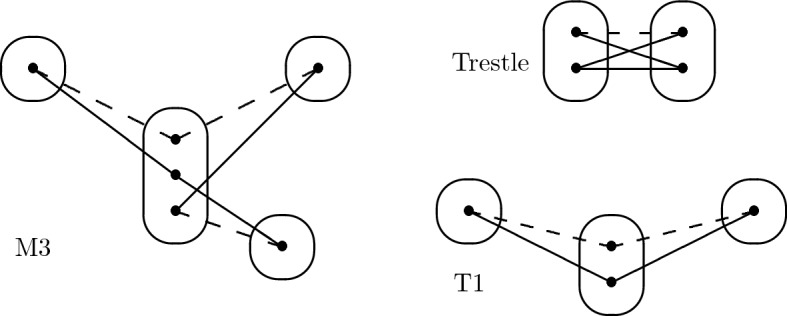
Patterns not solved by SAC

It is tedious but easy to verify that each of these instances has no solution and is singleton arc consistent. Any pattern which is solvable by SAC must therefore occur in each of these instances. Consider the patterns shown in Fig. 13. The patterns T1 and M3 do not occur in $I_{3,4}$. The pattern Trestle does not occur in $I_5$. It therefore follows that T1, M3 and Trestle are not solvable by SAC. Note that while M3 and Trestle are known to be NP-hard [17, 20], the pattern T1 is tractable (but not SAC-solvable, by the argument above) [20].

The *constraint graph of a patternP* with variables *X* is the graph $G=(X,E)$ such that $(x,y)\in E$ if *P* has a negative edge between variables $x,y \in X$.

### Proposition 1

**Fig. 14:**
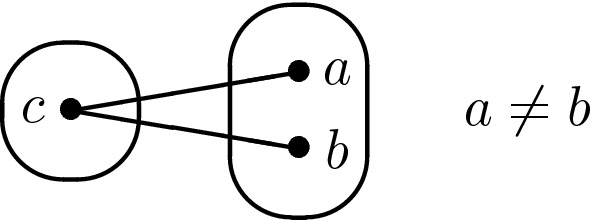
The pattern V

A monotone irreducible pattern *P* solvable by SAC satisfies:

1. None of the patterns T1, M3 and Trestle occur in *P*.
2. *P* has at most four variables.
3. *P* has at most one degree-3 variable and at most one non-trivial constraint in which the pattern V, shown in Fig. 14, occurs (with its centre point *c* at a variable with domain at size at most two), but does not have both a degree-3 variable and an occurrence of V. Furthermore, *P* has an acyclic constraint graph.
4. *P* has at most one negative edge per constraint, at most one point at which two negative edges meet (a negative meet point) and no point at which three negative edges meet. If *P* has a negative meet point, then none of its variables has domain size greater than two.

### Proof

The first property follows from the above discussion and the fact that *Q* occurs in *P* implies that CSP($\overline{\hbox {Q}}$) $\subseteq$ CSP($\overline{\hbox {P}}$).

Since a pattern *P* which is solvable by SAC must occur in $I_{4}^{3COL}$, *P* can have at most four variables. Since *P* is also a monotone pattern, no constraint of *P* contains only positive edges. Since *P* has at most four variables, either its constraint graph is connected or it is the union of two one-constraint patterns. In the latter case, by irreducibility and because Trestle cannot occur in *P*, *P* must be simply two negative edges between distinct variables (and hence all conditions of the proposition are trivially satisfied). So we assume in the rest of the proof that the constraint graph of *P* is connected. Since *P* has at most four variables, all of its variables are at a distance of at most three in its constraint graph.

We now consider the third property. By padding out $I_{4}^{3COL}$ with chains of equality constraints, it is easy to produce a SAC instance which has no solution and in which the pattern V does not occur in any non-trivial constraint at a distance of three or less from a degree-3 variable. It follows that no monotone irreducible pattern *P* with a degree-3 variable and in which the pattern V occurs is solvable by SAC. Using this same padding-with-equality argument, we can also deduce that in a monotone irreducible pattern *P* solvable by SAC: there is at most one degree-3 variable, there is at most one non-trivial constraint in which the pattern V occurs, and that *P* has no cycle in its constraint graph (the latter following from the fact that cycles of any *fixed* length can be eliminated from an instance by padding out with chains of equality constraints).

Finally, we consider the fourth property. Each inequality constraint $x_i \ne x_j$ in $I_{4}^{3COL}$ can be replaced by equivalent gadgets in which all constraints have at most one negative edge [17]. The resulting instance is still SAC. To be concrete, for each $i \in \{1,2,3,4\}$ and each $a \in \{1,2,3\}$, we create 21 new Boolean variables $x_{ia}^{r}$ ($r=0,1,\ldots ,20$) linked to the $x_i$ variables and between themselves by the following constraints: $(x_i=a) \Rightarrow x_{ia}^{0}$ and $x_{ia}^{r} \Rightarrow x_{ia}^{r+1}$ ($r=0,1,\ldots ,19)$. If $x_i$ is assigned the value *a*, then all the variables $x_{ia}^{r}$ must be assigned true. Each constraint $x_i \ne x_j$ ($1 \le i < j \le 4$) is then replaced by the chain of constraints $x_{ia}^{4j} \Rightarrow y_{ija}^{1}$, $y_{ija}^{1} \Rightarrow y_{ija}^{2}$, $y_{ija}^{2} \Rightarrow y_{ija}^{3}$, $y_{ija}^{3} \Rightarrow \overline{x_{ja}^{4i}}$, where $y_{ija}^{s}$ ($s=1,2,3$; $a=1,2,3$) are new boolean variables. In the resulting instance *I* there are no points at which three negative edges meet, no two negative meet points at a distance of three or less and no negative meet point at a distance of three or less from a variable with domain size three. We do not change the semantics of *I* (nor its singleton arc consistency) by replacing the constraints $(x_i=a) \Rightarrow x_{ia}^{0}$ by $(x_i=a) \Leftrightarrow x_{ia}^{0}$. In the resulting instance $I'$, no pattern V (Fig. 14) occurs with its centre point *c* at a variable with domain size greater than two. We can deduce that a monotone irreducible pattern *P* solvable by SAC (since it contains no 4-constraint path) has at most one negative edge per constraint, at most one negative meet point, no point at which three negative edges meet and the V pattern only occurs in *P* with its centre point *c* at a variable with domain of size at most two. Besides, *P* cannot have both a negative meet point and a variable with domain size three or more. $\square$

Proposition 1 allows us to narrow down monotone irreducible patterns solvable by SAC to a finite number, which we can summarize succinctly by the following proposition.

### Proposition 2

If *P* is a monotone irreducible pattern solvable by SAC, then *P* must occur in at least one of the patterns Q1,Q2,R1,$\ldots$,R10 (shown in Figs. 1 and 2).

### Proof

We saw in the proof of Proposition 1 that if *P* does not have a connected constraint graph, then *P* is simply the union of two negative edges: in this case *P* occurs in all the patterns R1,$\ldots$,R10. So we assume from now on that *P* has a connected constraint graph. From Proposition 1, we can deduce that the constraint graph of *P* is a either a star or a chain, with at most four vertices, and *P* has at most one negative edge per constraint. Such patterns must have one of the following four descriptions, which we analyse separately.

- *Phas a Single Degree-3 Variable* The constraint graph of *P* is necessarily a star. By Proposition 1, the pattern V does not occur in *P*. From this and the fact that *P* contains no dangling points and no mergeable points, we can deduce that each of the three degree-1 variables must have domain size 1. If the central degree-3 variable has domain size 3, then the fact that none of the patterns V, T1 and M3 occur in *P*, and that there are no mergeable points, implies that *P* must be the pattern Q1. If, on the other hand, the central variable has domain size 2, then since V and T1 do not occur in *P* and no three negative edges meet at a point, we can deduce that *P* must be Q2 (or a subpattern).
- *Pis of Degree 2 and has a Negative Meet Point* By Proposition 1, *P* has no domain of size greater than 2 and Trestle does not occur. It then follows by Proposition 1 and irreducibility of *P* that the pattern V cannot occur more than once (even in the same constraint). Since *P* has no dangling points, there are only four possible positions where V could occur. We can only add a limited number of positive edges without introducing T1, Trestle, dangling points or mergeable points. This gives rise to the four patterns R1,R2,R3,R4 (or subpatterns).
- *Pis of Degree 2, has no Negative Meet Point and All Domains Have Size 1 or 2* By the same argument as in the previous case, the pattern V can occur at most once. By the absence of Trestle and dangling points in *P*, and by symmetry, there are only two possible positions for the pattern V in *P*, if it occurs at all. Again, we can only add a limited number of positive edges without introducing T1, Trestle, dangling points or mergeable points. This gives rise to the three patterns R5,R6,R10 (or subpatterns).
- *Pis of Degree 2, has no Negative Meet Point and at Least One Size-3 Domain* The fact that *P* has no mergeable points and all variables have degree at most 2 implies that no domain can be greater than size 3. Indeed, from the fact that *P* is irreducible and that, by Proposition 1, no V can occur centred at a variable of domain size 3, we can deduce that there is exactly one variable with domain size 3. Adding positive edges to ensure that no two points are mergeable at this variable *v*, necessarily creates a V pattern. No other V can occur either in a different constraint (by Proposition 1) or in the same constraint otherwise we would have a V centred at *v* or Trestle would occur. Adding other positive edges, while satisfying the properties of Proposition 1, produces patterns R7,R8,R9 (or subpatterns). $\square$

## Conclusion

We have established SAC-solvability of five novel classes of binary CSPs defined by a forbidden pattern, three of which are generalisations of 2-SAT. For monotone patterns (defining classes of CSPs closed under removing constraints), there remains only a relatively small number of irreducible patterns whose SAC-solvability is still open. In addition to settling the remaining patterns, a possible line of future work is to study *sets* of patterns or partially-ordered patterns [23] that give rise to SAC-solvable (monotone) classes of CSPs.

## Appendix: SAC-solvability of T3

Recall T3 from Fig. 4. This is the only maximal two-constraints tractable pattern whose SAC-solvability is not determined by our main theorem, Proposition 1 and the results of [20].

### Theorem 6

CSP($\overline{\hbox {T3}}$) is solved by singleton arc consistency.

### Proof

Let $I \in$ CSP($\overline{\hbox {T3}}$) be a singleton arc consistent instance with no neighbourhood substitutable values. If T4 does not occur then *I* has a solution, so we examine the case where T4 occurs on variables (*x*, *y*, *z*) and values $a_x,a_y,b_y,c_y,a_z$ with $a_xa_y,a_xb_y,b_ya_z$ being positive edges and $a_xc_y,a_ya_z$ being negative edges. By arc consistency, $c_y$ has a support $b_x$ at *x* and because T3 does not occur $b_xb_y$ is a positive edge. Observe that $b_y$ dominates $c_y$ in *R*(*x*, *y*), and because neighbourhood substitutable values have been removed there must exist a variable *w* (possibly equal to *z*) and $b_w \in D(w)$ such that $b_yb_w$ is a negative edge and $c_yb_w$ is a positive edge. However, in this case T3 occurs on (*x*, *y*, *w*), so we obtain a contradiction. It follows that T4 cannot occur in the instance, and hence *I* has a solution. $\square$

## References

[1] Atserias, A., Bulatov, A., Dalmau, V.: On the power of k-consistency. In: Proceedings of the 34th International Colloquium on Automata, Languages and Programming (ICALP’07), pp. 279–290 (2007). 10.1007/978-3-540-73420-8_26

[2] Barto L, Kozik M (2014). Constraint satisfaction problems solvable by local consistency methods. J. ACM.

[3] Beldiceanu, N., Carlsson, M., Rampon, J.-X.: Global Constraint Catalog. http://sofdem.github.io/gccat/gccat/titlepage.html (2017)

[4] Berman J, Idziak P, Marković P, McKenzie R, Valeriote M, Willard R (2010). Varieties with few subalgebras of powers. Trans. Am. Math. Soc..

[5] Bessiere C, Debruyne R (2008). Theoretical analysis of singleton arc consistency and its extensions. Artif. Intell..

[6] Bessière C, Régin J, Yap RHC, Zhang Y (2005). An optimal coarse-grained arc consistency algorithm. Artif. Intell..

[7] Brailsford SC, Potts CN, Smith BM (1999). Constraint satisfaction problems: algorithms and applications. Eur. J. Oper. Res..

[8] Bulatov, A.: Bounded relational width. Unpublished manuscript (2009). http://www.cs.sfu.ca/~abulatov/papers/relwidth.pdf

[9] Bulatov A, Dalmau V (2006). A simple algorithm for Mal’tsev constraints. SIAM J. Comput..

[10] Bulatov A, Jeavons P, Krokhin A (2005). Classifying the complexity of constraints using finite algebras. SIAM J. Comput..

[11] Bulatov, A.A.: A dichotomy theorem for nonuniform CSPs. In: Proceedings of the 58th Annual IEEE Symposium on Foundations of Computer Science (FOCS’17), pp. 319–330 (2017). 10.1109/FOCS.2017.37

[12] Carbonnel, C., Cohen, D.A., Cooper, M.C., Živný, S.: On singleton arc consistency for CSPs defined by monotone patterns. In: Proceedings of the 35th Annual Symposium on Theoretical Aspects of Computer Science (STACS’18), pp. 19:1–19:15 (2018). 10.4230/LIPIcs.STACS.2018.19

[13] Chen H, Dalmau V, Gruien B (2013). Arc consistency and friends. J. Log. Comput..

[14] Cheng C-C, Smith SF (1997). Applying constraint satisfaction techniques to job shop scheduling. Ann. Oper. Res..

[15] Cohen DA, Cooper MC, Escamocher G, Živný S (2015). Variable and value elimination in binary constraint satisfaction via forbidden patterns. J. Comput. Syst. Sci..

[16] Cohen DA, Jeavons PG (2017). The power of propagation: when GAC is enough. Constraints.

[17] Cooper MC, Cohen DA, Creed P, Marx D, Salamon AZ (2012). The tractability of CSP classes defined by forbidden patterns. J. Artif. Intell. Res..

[18] Cooper MC, Cohen DA, Jeavons PG (1994). Characterising tractable constraints. Artif. Intell..

[19] Cooper MC, Duchein A, Mouelhi AE, Escamocher G, Terrioux C, Zanuttini B (2016). Broken triangles: from value merging to a tractable class of general-arity constraint satisfaction problems. Artif. Intell..

[20] Cooper MC, Escamocher G (2015). Characterising the complexity of constraint satisfaction problems defined by 2-constraint forbidden patterns. Discrete Appl. Math..

[21] Cooper MC, Jeavons PG, Salamon AZ (2010). Generalizing constraint satisfaction on trees: hybrid tractability and variable elimination. Artif. Intell..

[22] Cooper MC, Živný S (2011). Hybrid tractability of valued constraint problems. Artif. Intell..

[23] Cooper, M.C., Živný, S.: The power of arc consistency for CSPs defined by partially-ordered forbidden patterns. Log. Methods Comput. Sci. **13**(4) (2017). 10.23638/LMCS-13(4:26)2017

[24] Freuder EC (1982). A sufficient condition for backtrack-free search. J. ACM.

[25] Freuder, E.C.: Eliminating interchangeable values in constraint satisfaction problems. In: Proceedings of AAAI-91, pp. 227–233 (1991). http://www.aaai.org/Library/AAAI/1991/aaai91-036.php

[26] Grohe M (2007). The complexity of homomorphism and constraint satisfaction problems seen from the other side. J. ACM.

[27] Idziak P, Marković P, McKenzie R, Valeriote M, Willard R (2010). Tractability and learnability arising from algebras with few subpowers. SIAM J. Comput..

[28] Kozik, M.: Weak consistency notions for all the CSPs of bounded width. In: Proceedings of the 31st Annual ACM/IEEE Symposium on Logic in Computer Science (LICS’16), ACM, pp. 633–641 (2016). 10.1145/2933575.2934510

[29] Lim, N., Majumdar, S., Ashwood-Smith, P.: A constraint programming-based resource management technique for processing MapReduce jobs with SLSs on clouds. In: Proceedings of the 43rd International Conference on Parallel Processing (ICPP’14), IEEE, pp. 411–421 (2014). 10.1109/ICPP.2014.50

[30] Mohr R, Henderson TC (1986). Arc and path consistency revisited. Artif. Intell..

[31] Ozturk C, Ornek MA (2016). Optimisation and constraint based heuristic methods for advanced planning and scheduling systems. Int. J. Ind. Eng. Theory Appl. Pract..

[32] Rossi F, Van Beek P, Walsh T (2006). Handbook of Constraint Programming.

[33] Senkul P, Toroslu IH (2005). An architecture for workflow scheduling under resource allocation constraints. Inf. Syst..

[34] Zhuk, D.: A proof of CSP dichotomy conjecture. In: Proceedings of the 58th Annual IEEE Symposium on Foundations of Computer Science (FOCS’17), pp. 331–342 (2017). 10.1109/FOCS.2017.38

